# Response mechanisms to acid stress promote LF82 replication in macrophages

**DOI:** 10.3389/fcimb.2023.1255083

**Published:** 2023-10-10

**Authors:** Ting Yao, Yu Huang, Zimeng Huai, Xingmei Liu, Xiaowen Liu, Yutao Liu, Hao Sun, Yu Pang

**Affiliations:** ^1^ TEDA Institute of Biological Sciences and Biotechnology, Nankai University, Tianjin, China; ^2^ The Key Laboratory of Molecular Microbiology and Technology, TEDA Institute of Biological Sciences and Biotechnology, Nankai University, Ministry of Education, Tianjin, China; ^3^ Academy of Psychology and Behavior, Faculty of Psychology, Tianjin Normal University, Tianjin, China

**Keywords:** adherent-invasive *E. coli* (AIEC), macrophages, acid shock, acid fitness, nitrate utilization, flagellar

## Abstract

**Background:**

Adherent–invasive *E. coli* (AIEC) LF82 is capable of adhering to and invading intestinal epithelial cells, as well as replicating within macrophages without inducing host cell death.

**Methods:**

We compared the transcriptomics of LF82 at pH=7.5 and pH=5.8 by RNA-sequencing, and qRT-PCR verified differentially expressed genes (DEGs). The deletion mutants of DEGs in the treatment group (pH=5.8) compared to the control group (pH=7.5) were constructed by λ recombinant. The replication differences between the mutants and WT infected Raw 264.7 at 24 h.p.i were analyzed by combining LB solid plate count and confocal observation. NH_4_Cl and chloroquine diphosphate (CQ) were used for acid neutralization to study the effect of pH on the replication of LF82 in macrophages. Na_2_NO_3_ was added to RPMI 1640 to study the effect of nitrate on the replication of LF82 in macrophages. 0.3% solid LB was used for flagellar motility assay and Hela was used to study flagellar gene deletion mutants and WT adhesion and invasion ability.

**Results:**

In this study, we found that infection with LF82 results in acidification of macrophages. Subsequent experiments demonstrated that an intracellular acidic environment is necessary for LF82 replication. Transcriptome and phenotypic analysis showed that high expression of acid shock genes and acid fitness genes promotes LF82 replication in macrophages. Further, we found that the replication of LF82 in macrophages was increased under nitrate treatment, and nitrogen metabolism genes of LF82 were upregulated in acid treatment. The replication in macrophages of *ΔnarK, ΔnarXL, ΔnarP*, and *Δhmp* were decreased. In addition, we found that the expression of flagellar genes was downregulated in acidic pH and after LF82 invading macrophages. Motility assay shows that the movement of LF82 on an acidic semisolid agar plate was limited. Further results showed that ΔfliC and ΔfliD decreased in motility, adhesion ability, and invasion of host cells, but no significant effect on replication in macrophages was observed.

**Conclusion:**

In this study, we simulated the acidic environment in macrophages, combined with transcriptome technology, and explained from the genetic level that LF82 promotes replication by activating its acid shock and fitness system, enhancing nitrate utilization, and inhibiting flagellar function.

## 1 Introduction

Inflammatory bowel disease (IBD) is a chronic disease with unknown etiology that results in uncontrolled inflammation of the gastrointestinal tract (Kaplan, 2015; Vindigni et al., 2016; Wark et al., 2021). Historically, IBD has been regarded as a common intestinal disorder in developed countries. However, with changing epidemiological trends in the 21st century, IBD has become a global health concern. Currently, it is estimated that the worldwide prevalence of IBD approaches 90 cases per 100,000 individuals (Kofla-Dłubacz et al., 2022). IBD can be further classified as Crohn’s disease (CD) and ulcerative colitis (UC) based on clinical manifestations and intestinal localization. CD is a transmural inflammatory disease that affects the entire small intestine and colon, while UC is characterized by mucosal inflammation limited to the colon (Feldman et al., 2020).

Previous research has indicated that individuals with CD exhibit abnormal changes in the composition of their intestinal microbiota including a significant reduction in *Clostridium* and *Bacteroides* populations and an increase in Enterobacteriaceae. Notably, a specific pathogenic group of *Escherichia coli*, named adherent–invasive *E. coli* (AIEC), has been extensively linked to CD (Darfeuille-Michaud et al., 2004; Martin et al., 2004; Baumgart et al., 2007; Frank et al., 2007; Knights et al., 2013; Rooks et al., 2014; Vich Vila et al., 2018). AIEC is capable of penetrating the epithelial barrier, surviving, and replicating in macrophages in CD patients. Further investigation has revealed that the classic AIEC LF82 can invade macrophages and establish a replication niche by assembling biofilm-like communities which protect it from phagolysosomal attack (Prudent et al., 2021). However, the precise mechanism by which LF82 replicates within macrophages remains insufficiently elucidated.

The pH in macrophages rapidly decreases when infected by bacteria. Studies have reported that an acid environment is necessary for LF82 to survive and replicate within macrophages (Bringer et al., 2006, 2012). In *E. coli*, multiple genes have been shown to be associated with acid tolerance. The acid shock protein Asr, encoded by the *asr* gene, strongly supports the growth of *E. coli* at moderate acidity conditions (Ramos-Morales, 2012), and the acid fitness island (AFI) plays a role in the acid response (Mates et al., 2007). Meanwhile, the periplasmic chaperones HdeA and HdeB are crucial for bacterial survival at low pH in *E. coli* and *Shigella* spp. (Kern et al., 2007; Carter et al., 2012). However, the mechanism of LF82 survival and replication in macrophages in response to acid stress still unclear.

Nitrate (NO^3-^) and nitrite (NO^2-^) serve as crucial nitrogen sources for both host and pathogen. When macrophages are activated by bacterial lipopolysaccharide, nitrite and nitrate are synthesized (Stuehr and Marletta, 1985; Iyengar et al., 1987). Nitrogen metabolism within a restricted niche in macrophages is key to the survival and pathogenesis of intracellular pathogens (Borah et al., 2019). For instance, *Salmonella* Typhimurium utilizes nitrate as an electron acceptor to facilitate its growth during intestinal infection and enhance systemic virulence (Li et al., 2022). Several genes have been demonstrated to be crucial for nitrate metabolism including *hmpA*, which encodes the flavohemoglobin protein that catalyzes the conversion of NO and O_2_ to nitrate (Stevanin et al., 2002); *narK*, the primary transporter for nitrate and nitrite (Clegg et al., 2002; Jia and Cole, 2005; Clegg et al., 2006); and *narXL*, a two-component system responsible for sensing nitrate (Noriega et al., 2010; Durand and Guillier, 2021). However, the effect of nitrogen metabolism of LF82 in macrophages remains unknown.

Flagella is an important motor macromolecular machine and virulence factor of bacteria (Sun et al., 2022). Flagella allow bacteria to actively move toward favorable environments and away from hazardous areas in order to reach and settle in new habitats (Colin et al., 2021; Thormann et al., 2022). The motility and biosynthesis of flagella are strictly regulated by three classes of genes (Chilcott and Hughes, 2000; Frye et al., 2006) and affected by diverse environmental signals such as mucin, temperature, and pH (Sun et al., 2022). The Class I gene *flhDC* encodes the master flagellar regulator, with FlhD and FlhC forming a heterotetramer (FlhD4C2)(Khan et al., 2020). Class II genes, including *fliFGHIJK*, *fliLMNOPQR*, *fliE*, *flhBAE*, *flgBCDEFGHIJ*, *fliAZY*, and *flgAMN*, encode structural and assembly proteins required for the biosynthesis of the hook-basal body and a pair of flagellar regulatory proteins, FliA and FlgM (Kutsukake et al., 1990; Kalir et al., 2001). Class III genes, including *flgKL*, *fliDST*, *flgMN*, *fliC*, *tar-tap-cheRBYZ*, and *motAB-cheAW*, encode cell-distal structural components of the flagellum and flagellar function (rotation and chemotaxis) (Osterman et al., 2015; Khan et al., 2020). The acid signal of the host can suppress flagellar biosynthesis and motility of *Salmonella* to avoid recognition by the host immune system (Wang et al., 2022). Meanwhile, *Salmonella* turns off flagellar biosynthesis to promote intracellular replication and cause systemic disease (Ma et al., 2021).

To elucidate the mechanism by which an acidic environment facilitates LF82 intracellular replication in macrophages, RNA-Seq analysis was performed to investigate the global impact of acidity on gene expression. Transcriptome analysis of LF82 under acidic conditions revealed that the expression of genes involved in acid tolerance and nitrate transport/metabolism was upregulated, while the expression of flagellar genes was downregulated. Acid regulatory genes play a pivotal role in LF82’s intracellular replication within macrophages. The utilization of nitrate by LF82 provides energy for its survival and replication within macrophages. Additionally, the closure of flagellar function limits LF82’s movement and conserves energy, which further facilitates its replication within macrophages. Our study reveals the mechanism by which LF82 utilizes an acidic environment to replicate within macrophages.

## 2 Results

### 2.1 An acidic environment is required for the intracellular replication of LF82

**Figure 1:**
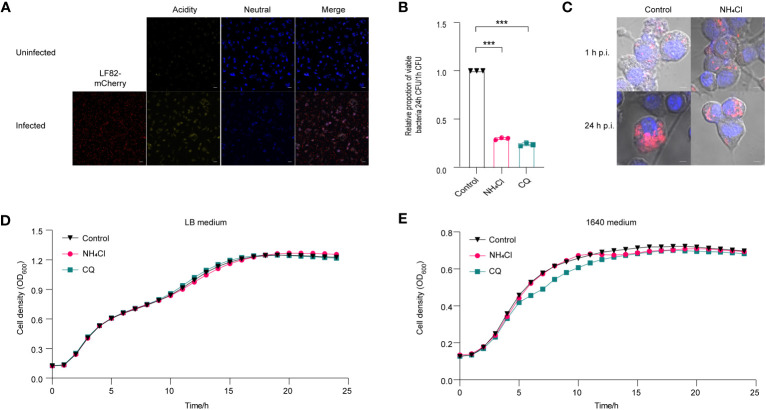
Effect of acidic pH on the replication of LF82 in macrophages. **(A)** The pH variation between uninfected macrophages and LF82-infected macrophages was indicated by LysoSensor™ Yellow/Blue. **(B)** The intracellular replication of LF82 in macrophages and macrophages treated with 30 mM NH_4_Cl or 10 µM CQ. **(C)** Confocal observation of LF82 (red) intracellular replication in macrophages and macrophages treated with 30 mM NH_4_Cl or 10 µM CQ at indicated time point. **(D)** Growth curve of LF82 treated with 30 mM NH_4_Cl or 10 µM CQ in LB medium. **(E)** Growth curve of LF82 treated with 30 mM NH_4_Cl or 10 µM CQ in 1640 medium. Data were obtained from three independent experiments and analyzed using Student’s *t*-test. ***P < 0.001.

An acidic environment is essential for LF82 intracellular replication within macrophage phagolysosomes (Bringer et al., 2006). To monitor pH fluctuations in macrophages, we employed LysoSensor, which emits blue and yellow fluorescence (yellow fluorescence indicates pH<7, stronger yellow fluorescence reflects a lower pH level) to compare pH variations between uninfected and LF82-infected macrophages. The result showed that infected macrophages exhibited a yellow hue in comparison to uninfected cells (**Figure 1A**), indicating a rapid decline in pH levels upon LF82 infection. This finding is consistent with previous reports suggesting that intracellular acidification results from LF82 replication within macrophages(Bringer et al., 2012).

NH_4_Cl and chloroquine diphosphate (CQ) have been demonstrated to effectively neutralize the acidic pH in macrophages (Bringer et al., 2006; Bringer et al., 2012). A replication assay demonstrated that neutralization of acidic environment in macrophages with 30 mM NH_4_Cl or 10 μM CQ resulted in a reduction of LF82 replication by 3.3-fold and 4.2-fold, respectively (**Figure 1B**). Meanwhile, confocal scanning revealed a significant decrease in bacterial load within macrophages treated with NH_4_Cl and CQ at 24 h post-infection (p.i.) compared to the untreated control group (**Figure 1C**). An *in vitro* growth curve demonstrated that 30 mM NH_4_Cl or 10 µM CQ had no impact on LF82 proliferation in LB or RPMI 1640 medium, indicating that the reduction of LF82 replication in macrophages treated with either NH_4_Cl or CQ was not due to the growth defect of LF82 in LB and 1640 medium (**Figures 1D, E**). These results indicate that the acidic microenvironment within macrophages facilitates LF82 replication.

### 2.2 Transcriptome responses of LF82 under acid environment

**Figure 2:**
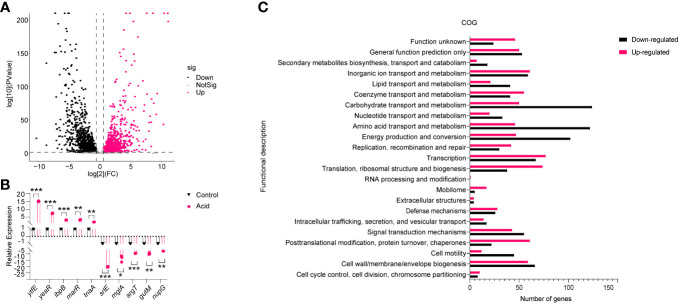
Analysis of the gene expression of LF82 under acid treatment. **(A)** Volcano plot depicts differentially expressed genes in transcriptome. **(B)** Verification of differentially expressed genes by qRT-PCR. **(C)** Clusters of orthologous groups (COG) analysis of acid-treated DEGs in LF82. Data were obtained from three independent experiments and analyzed using Student’s *t*-test. *P < 0.05, **P < 0.01, ***P < 0.001.

To investigate the mechanism underlying LF82 replication in an acid environment, we simulated the pH of LF82 in acidic macrophages (Bringer et al., 2005; Bringer et al., 2007), and the transcriptome of LF82 cultured in a pH 5.8 acid environment for 30 min was analyzed. After filtering low-quality reads, a total of 24,815,427 and 18,614,200 reads were obtained from the acid-treated and control group, respectively. Approximately 97.22% of the total reads for the acid treated group and 98.31% of those for the control group were uniquely mapped to the reference genome. Subsequently, gene expression profiles were compared. The results showed that a total of 1,997 genes were differentially expressed in the acid-treated group compared to those in the control group. Of these, 995 and 1002 genes were categorized as upregulated and downregulated, respectively (fold change > 2 and *P* value < 0.05), show as **Table S1** and the volcano plot of DEGs reported in **Figure 2A**. To verify the transcriptome results, 10 differentially expressed genes (DEGs) were randomly selected for qRT-PCR analysis. The results showed that the expression patterns of these DEGs were consistent with those of RNA-Seq data, indicating the robustness and validity of our RNA-Seq approach (**Figure 2B**).

Subsequently, DEGs in the acid treated and control group were classified using the NCBI COG functional categories annotation system. The COG categories significantly enriched in the group of upregulated genes were primarily associated with post-translational modification, protein turnover, chaperones, mobilome, translation, ribosomal structure, and biogenesis. The COG categories significantly enriched in the list of downregulated genes included carbohydrate transport and metabolism, amino acid transport and metabolism, energy production, and conversion (**Figure 2C**). These results indicate that the impact of acid conditions on LF82 gene expression is bidirectional, which provides a basis for us to study the replication mechanism of LF82 in acid macrophages from the transcriptional level.

### 2.3 Acid regulatory genes affect intracellular replication of LF82

**Figure 3:**
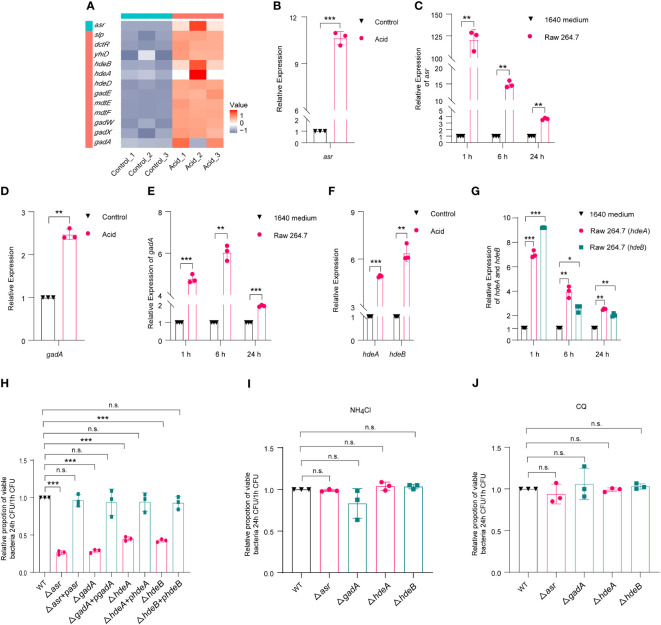
*E*. *coli* acid shock and fitness genes affect the replication of LF82 in macrophages. **(A)** Heat map of acid fitness genes expression of LF82 under acid treatment. **(B)** qRT-PCR detected the expression of *asr* in LF82 under acid treatment. **(C)** qRT-PCR detected the expression of *asr* in LF82-infected macrophages at indicated time point. **(D)** qRT-PCR detected the expression of *gadA* in LF82 under acid treatment. **(E)** qRT-PCR detected the expression of *gadA* in LF82-infected macrophages at indicated time point. **(F)** qRT-PCR detected the expression of *hdeA* and *hdeB* in LF82 under acid treatment. **(G)** qRT-PCR detected the expression of *hdeA* and *hdeB* in LF82-infected macrophages at indicated time point. **(H)** Comparison of intracellular replication between Δ*asr*, Δ*asr+*p*asr*, Δ*gadA*, Δ*gadA+*p*gadA*, Δ*hdeA*, Δ*hdeA+*p*hdeA*, Δ*hdeB*, Δ*hdeB+*p*hdeB* and WT at 24 h p.i. **(I)** Comparison of intracellular replication between Δ*asr*, Δ*asr+*p*asr*, Δ*gadA*, Δ*gadA+*p*gadA*, Δ*hdeA*, Δ*hdeA+*p*hdeA*, Δ*hdeB*, Δ*hdeB+*p*hdeB* and WT at 24 h p.i. treated with 30 mM NH_4_Cl. **(J)** Comparison of intracellular replication between Δ*asr*, Δ*asr+*p*asr*, Δ*gadA*, Δ*gadA+*p*gadA*, Δ*hdeA*, Δ*hdeA+*p*hdeA*, Δ*hdeB*, Δ*hdeB+*p*hdeB* and WT at 24 h p.i. treated with 10 μM CQ. Data were obtained from three independent experiments and analyzed using Student’s *t*-test. *P < 0.05, **P < 0.01, ***P < 0.001; n.s., not significant.

Based on RNA-Seq results, the expression of *asr* (acid shock gene) was significantly upregulated, by 622-fold, in the acid-treated group compared to the control group (**Figures S2A**, **3A**). RT-qPCR results further verified that *asr* was 10.63-fold upregulated in the acid-treated group compared to the control group (**Figure 3B**). Meanwhile, the expression of *asr* was significantly upregulated, by 128.69-fold, in intra-macrophage LF82 at 1 h p.i. and maintained a high level of expression at 6 h (14.85 fold) and 24 h (3.63 fold) p.i., respectively (**Figure 3C**). We further generated *asr* mutant (Δ*asr*) to evaluate whether *asr* affects the intracellular replication of LF82. The results showed that Δ*asr* led to a 3.85-fold decrease in LF82 replication ability within macrophages, these differences could be restored to wild-type levels when complementary plasmids pSWK129-*asr* was introduced into the Δ*asr* mutant (**Table S3**; **Figure 3H**), indicating that *asr* promotes the replication of LF82 in macrophages. However, there was no difference in the replication abilities between WT and Δ*asr* in macrophages which neutralized the acid condition with NH_4_Cl or CQ (**Table S3**, **Figures 3I, J**). These results indicated that *asr* plays an important role in survival and replication of LF82 in macrophages under acidic conditions.

In addition, the volcano map and heat map results showed that the expression of several acid fitness genes was also upregulated in the LF82 acid-treated group compared to the control group (**Figures S2A**, **3A**). Further qRT-PCR results confirmed the upregulation of *gadA*, *hdeA*, and *hdeB* (**Figures 3D, F**). To investigate the expression of *gadA* in LF82-infected macrophages at 1 h, 6 h, and 24 h p.i., qRT-PCR was performed. The results showed that *gadA* was upregulated by 4.77-fold, 6.03-fold, and 1.93-fold in LF82 infected macrophages at 1 h, 6 h, and 24 h p.i., respectively (**Figure 3E**). Additionally, the expression of *hdeA* in LF82-infected macrophages was upregulated by 7.02-fold, 3.96-fold, and 2.53-fold at 1 h, 6 h, and 24 h p.i., respectively (**Figure 3G**). Furthermore, *hdeB* exhibited a significant increase in expression levels, 9.20-fold at 1 h p.i., followed by increases of 2.66-fold and 2.05-fold at 6 h and 24 h p.i. (**Figure 3G**). These results suggest that *gadA*, *hdeA*, and *hdeB* play an important role after LF82 infects macrophages. To identify the effect of *gadA*, *hdeA*, and *hdeB* in LF82 intracellular replication, we generated Δ*gadA*, Δ*hdeA*, and Δ*hdeB*. As shown in **Table S3** and **Figure 3H**, the replication ability of Δ*gadA* in macrophages was reduced by 3.57-fold compared to that of WT, complementary strains of Δ*gadA*+p*gadA* could recover the replication ability of WT, indicating that *gadA* is required for the replication of LF82 in macrophages. When the acidic pH of macrophages was neutralized by NH_4_Cl and CQ, the replication ability of WT in macrophages was significantly reduced, while there was no significant difference in the replication ability between WT and Δ*gadA* in untreated and acid-neutralized macrophages (**Figures 3I, J**). In addition, Δ*hdeA* and Δ*hdeB* shared silimiar trends with Δ*gadA* (**Table S3**; **Figures 3H–J**). These findings suggest that acid regulatory genes play crucial roles in the survival and replication of LF82 within macrophages.

In addition, the results of the growth curve showed that there was no difference between gene deletion mutants (Δ*asr*, Δ*gadA*, Δ*hdeA* and Δ*hdeB*) and WT in control 1640 medium. But there was significant difference between gene deletion mutants and WT when growth in 1640 medium (pH=5.8) (**Figures S3A, S3B**). These results further indicated that *asr*, *gadA*, *hdeA* and *hdeB* plays an important role in survival and replication of LF82 in macrophages under acidic conditions.

### 2.4 The acidic environment promotes nitrate metabolism and enhances intracellular replication

**Figure 4:**
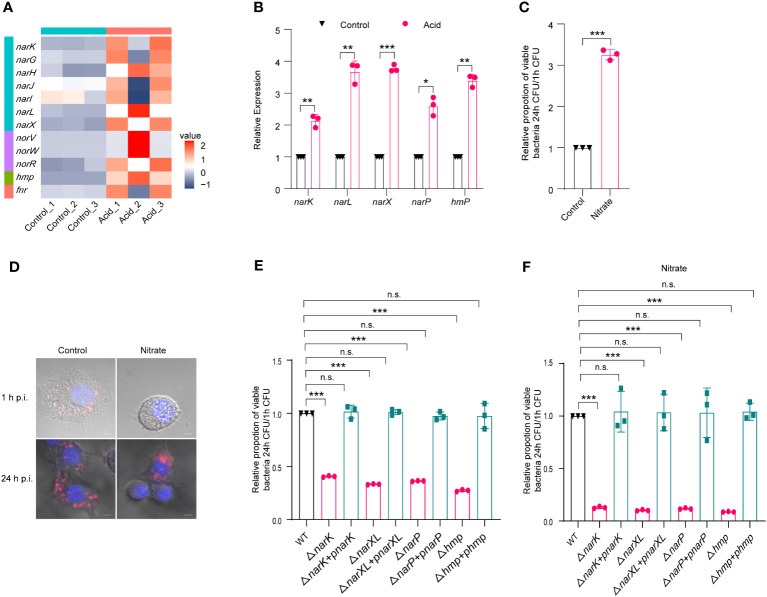
The metabolism of nitrate by LF82 was beneficial to intracellular replication. **(A)** Heat map of nitrate metabolism genes of LF82 under acid treatment. **(B)** qRT-PCR verified the expression level of *narK*, *narL*, *narX*, *narP*, and *hmp* genes in transcriptome. **(C)** Effect of 0.3 mM Na_2_NO_3_ on replication of LF82 in macrophages. **(D)** Confocal observation LF82 intracellular replication in macrophages treated with or without 0.3 mM Na_2_NO_3_. **(E)** Comparison of intracellular replication between *narK*, Δ*narK+*p*narK*, *narXL*, Δ*narXL+*p*narXL*, *narP*, Δ*narP+*p*narP*, *hmp*, Δ*hmp+*p*hmp* and WT. **(F)** Comparison of intracellular replication between *narK*, Δ*narK+*p*narK*, *narXL*, Δ*narXL+*p*narXL*, *narP*, Δ*narP+*p*narP*, *hmp*, Δ*hmp+*p*hmp* and WT treated with or without 0.3 mM Na_2_NO_3_. Data were obtained from three independent experiments and analyzed using Student’s *t*-test. *P < 0.05, **P < 0.01, ***P < 0.001; n.s., not significant.

Transcriptome analysis showed that several nitrate-metabolism-related genes were upregulated, including *narGHIJ* gene cluster, *narK*, nitrate sensing two-component system *narX*/*narL*, and *hmp*, as shown in the volcano plot and heat map (**Figures S2A**, **4A**). The upregulation of genes involved in nitrogen metabolism, namely, *narK*, *narL*, *narX*, *narP*, and *hmp*, was confirmed by qRT-PCR analysis following acid treatment, with fold changes of 2.13-, 3.67-, 3.77-, 2.59- and 3.39-fold, respectively (**Figure 4B**). As replication within host macrophages of LF82 is essential for chronic inflammation, the impact of nitrate utilization on LF82 replication in macrophages was assessed. The addition of 0.3 mM Na_2_NO_3_ to the 1640 medium resulted in a significant increase (3.25-fold) in LF82 replication within macrophages (**Figure 4C**). Meanwhile, confocal scanning results demonstrated that Na_2_NO_3_ treatment significantly increases bacterial quantity in macrophages compared to the untreated group at 24 h p.i. (**Figure 4D**), indicating a potential role of nitrate metabolism in promoting LF82 replication within macrophages.

Furthermore, we generated mutants lacking *narK* (Δ*narK*), *narXL* (Δ*narXL*), *narP* (Δ*narP*), and *hmp* (Δ*hmp*), which are genes related to nitrate metabolism. Δ*narK*, Δ*narXL*, Δ*narP*, and Δ*hmp* resulted in a significant reduction in LF82 replication within macrophages (**Table S4**; **Figures 4E**). In addition, the gene deletion strains Δ*narK*, Δ*narXL*, Δ*narP* and Δ*hmp* lost the ability to enhance nitrate metabolism and then enhance replication ability in macrophages (**Table S4**; **Figures 4F**). These differences could be restored to wild-type levels by Δ*narK+*p*narK*, Δ*narXL+*p*narXL*, Δ*narP+*p*narP*, and Δ*hmp+*p*hmp* complementary strains (**Table S4**; **Figures 4E, F**), indicating that *narK*, *narXL*, *narP* and *hmp* affected the replication of LF82 in macrophages by influencing the metabolism of nitrate.

In addition, the results of the growth curve showed that there was no difference between gene deletion mutants (Δ*narK*, Δ*narXL*, Δ*narP* and Δ*hmp*) and WT in control 1640 medium and 1640 medium (pH=5.8) (**Figures S4A, S4B**). These results further indicated that *narK*, *narXL*, *narP* and *hmp* did not affect the growth of LF82 in macrophages under acidic conditions.

### 2.5 Acidic conditions restrict the motility of LF82 to its intracellular replication.

**Figure 5:**
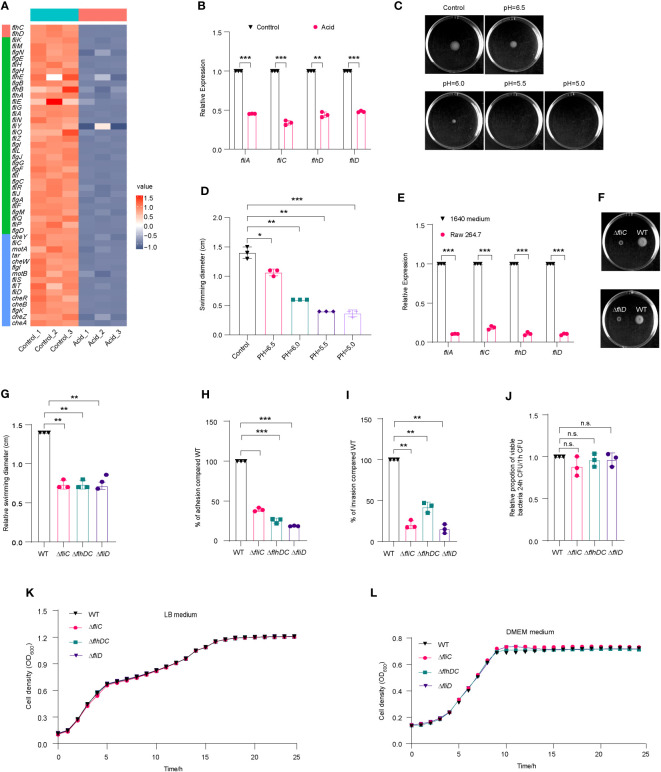
Flagella movement of LF82 was limited under acid treatment and in macrophages. **(A)** Heat map of flagellar genes expression in transcriptome of LF82 under acid treatment. **(B)** qRT-PCR verified the expression level of *fliA*, *fliC*, *flhD*, and *fliD* genes in transcriptome. **(C)** The motion of LF82 under different pH semisolid mediums. **(D)** The diameter of swimming circles of LF82 under different pH semisolid mediums. **(E)** Expression of LF82 flagellar genes in macrophages compared to culture in 1640 medium at 1 h p.i. **(F)** The motion of Δ*fliC* and Δ*fliD* compared to that of WT on semisolid medium. **(G)** The diameter of swimming circles of Δ*fliC* and Δ*fliD* compared that of WT on semisolid medium. **(H)** Adhesion of Δ*fliC* and Δ*fliD* into epithelial cells compared to that of WT at 3 h p.i. **(I)** Internalization of Δ*fliC* and Δ*fliD* into epithelial cells compared to that of WT at 4 h p.i. **(J)** Replication ability of Δ*fliC* and Δ*fliD* in macrophages compared to that of WT. **(K)** Growth curve of *fliC*, *fliD* and WT in LB medium. **(L)** Growth curve of *fliC*, *fliD* and WT in DMEM medium. Data were obtained from three independent experiments and analyzed using Student’s *t*-test. *P < 0.05, **P < 0.01, ***P < 0.001; n.s., not significant.

Overexpression of flagellar genes have been found to attenuate the virulence of *Salmonella*, while suppressing flagellar expression in acidic environments facilitates intracellular replication within macrophages (Yang et al., 2012). The analysis of transcriptome data revealed a significant downregulation of flagellar genes in the acid-treated group (**Figure 5A**), which was further confirmed by qRT-PCR (**Figure 5B**). The motility of LF82 in semisolid media was significantly impaired at pH levels below 6, with a reduction in swimming ability observed particularly at pH values lower than 5.5, where the diameter of swimming decreased to approximately 0.4-fold compared to that seen under neutral conditions (**Figures 5C, D**). These results suggest that acidic environments may limit flagellar formation and movement.

We also compared the expression levels of flagellar genes of LF82 cultured in 1640 medium and infected macrophages using qRT-PCR. At 1 h p.i., *fliA*, *flhD*, and *fliD* were downregulated by approximately 10-fold, while *fliC* was downregulated by approximately 5-fold (**Figure 5E**). These results suggest that the decreased expression of LF82 flagellar genes in macrophages is associated with the acidic environment within host cells.

We obtained Δ*fliC* and Δ*fliD* mutants; these displayed a reduced swimming ability of 0.7-fold and 0.7-fold, respectively, compared to WT (**Figures 5F, G**). The adhesion abilities of Δ*fliC* and Δ*fliD* to epithelial cells were reduced to 39.1% and 18.6% compared to WT, respectively (**Figures 5H**). The invasion abilities of Δ*fliC* and Δ*fliD* to epithelial cells were reduced to 20.2% and 15.2% compared to WT, respectively (**Figure 5I**). However, there was no significant difference of intracellular replication abilities within macrophages between Δ*fliC*, Δ*fliD*, and WT (**Figure 5J**). These findings suggest that the regulation of flagellar expression by *fliC* and *fliD* may contribute to the motility, adhesion, and invasion ability of LF82 in epithelial cells. However, deletion of these genes did not significantly affect LF82 replication in macrophages.

Shown as **Figures 5K, L**, there was no difference between *fliC*, *fliD* and WT in LB medium and DMEM medium. These results indicated that *fliC* and *fliD* reduced swimming ability of LF82 and reduced the adhesion and invasion ability to Hela cells, not because the growth was affected by the deletion of fliC and fliD genes.

In addition, the results of the growth curve showed that there was no difference between gene deletion mutants (Δ*fliC* and Δ *fliD*) and WT in control 1640 medium and 1640 medium (pH=5.8) (**Figures S5A, S5B**). These results further indicated that *narK*, *narXL*, *narP* and Δ*hmp* did not affect the growth of LF82 in macrophages under acidic conditions.

## 3 Discussion

CD is a chronic relapsing IBD potentially affecting any portion of the gastrointestinal tract from the mouth to the anus, with increasing incidence worldwide (Laass et al., 2014). It is critical to diagnosis and manage CD because of the threat to human health and the economic damage caused by CD. Macrophages in CD patients are unable to effectively control the replication of CD-associated LF82 strain, which is an important factor that leads to persistent chronic inflammation (Lapaquette et al., 2012; Vazeille et al., 2015; Buisson et al., 2019). However, the specific mechanism of how LF82 survives and replicates in macrophages remains unclear.

According to the results of genomic phylogenetic tree analysis, LF82 is the closest relative to neonatal meningitis *E. coli* (NMEC). During NMEC invasion human brain microvascular endothelial cells (HBMECs) histone-like nucleoid structuring protein (H-NS) senses the acidic pH within endosomes to de-repress *ybdO* transcription, resulting in increased YbdO-dependent K1 capsule synthesis which enhances the survival of NMEC in HBMECs and facilitates the virulence of NMEC (Fan et al., 2022). The acidic pH in macrophages downregulates the expression of AsiR in *Salmonella* typhimurium, resulting in the downregulation of the expression of flagellar gene, promoting the intracellular replication and systemic infection of *Salmonella* typhimurium (Ma et al., 2021). Wanwu Li et al. reported the mechanism of cytoplasmic acidification through transport and use of nitrate in *Salmonella* typhimurium, which ultimately promotes the intracellular replication and systemic infection of *Salmonella* Typhimurium (Li et al., 2022). In this study, we found that LF82 infection resulted in macrophages acidifying rapidly. Further results verified that acid shock gene *asr* and acid fitness island genes were significantly upregulated under acidic conditions and in macrophages, and the acidic environment of macrophage is necessary for LF82’s survival and replication. Combined with the reported studies of other intracellular pathogens, it suggested that intra-macrophage acidification is important for the survival and virulence of these intracellular pathogens.

Nitrogen metabolism is one of the important mechanisms for bacteria to adapt to an intracellular acid environment. Nitrate utilization, which is activated by both the global regulator Fnr and the nitrate-sensing two-component system NarX/NarL in *S.* Typhimurium, promotes *S.* Typhimurium intracellular replication and systemic pathogenicity (Li et al., 2022). In this study, we found that nitrogen-metabolism-related genes of LF82 were upregulated in acid conditions. Deletion of nitrate metabolism genes weakened the nitrate utilization and intracellular replication capacity of LF82. These results imply that the nitrogen metabolism of LF82 may contribute to its adaptation to the macrophages’ environment and promote LF82 intra-macrophage replication.

Bacterial flagella constitute a macromolecular machine (Duan et al., 2013) that is vital for bacterial survival in different environments (Tan et al., 2021) and essential for its motility, niche colonization, and pathogenesis (Subramanian and Kearns, 2019). Flagellar biosynthesis is an energy-intensive process requiring 2% of the cell’s biosynthetic resources, and flagellar rotation consumes 0.1% of the cell’s energy (Fontaine et al., 2008). Bacteria remove their flagella under starvation in a programmed way to reduce the significant energy burden (Zhu and Gao, 2020). We found that the acid environment inhibited flagellar genes’ expression, and that flagellar genes were downregulated after LF82 invaded macrophages. This suggests that LF82 shuts down flagellar biosynthesis to help it reduce unnecessary energy loss in order to guarantee bacterial intracellular survival.

Compared with the control group, a total of 1997 differentially expressed genes were identified in the acid treated group, including 995 upregulated and 1002 downregulated genes. This suggests that there are many more genes that change besides the acid, nitrogen metabolism, and flagellar genes we found above. For example, the operon for the sorbitol P-enolpyruvate phosphotransferase transport system (*srlA*, *srlE*, *srlB*, *srlD*, *gutM*, and *srlR*) of LF82 was downregulated significantly. This suggests that carbohydrate metabolism of LF82 may be affected by the acidic environment, thus affecting the survival and replication of LF82 in acid macrophages. Thus, the metabolic characteristics of LF82 in acidic pH macrophages require further study. Additionally, a bacterial two-component system can sense environmental signals, such as chemical signals, temperature, pressure, etc., from the outside of the bacteria, and regulate internal cell signals through kinase reactors, to adjust the bacteria to produce adaptive responses. The upregulated expression of *soxS* and *soxR* indicates that an acid signal activates the redox factor of LF82, which may help LF82 resist oxidative damage in macrophages. A two-component system, RstA/RstB, known to regulate biofilm formation, nitrogen metabolism, spore formation, and contribute to bacterial intracellular replication and virulence is upregulated, suggesting that the acidic environment of macrophages may help LF82 replicate in macrophages.

Overall, in this study we revealed that a macrophage’s acid environment is necessary for LF82 intracellular replication. First, acid shock protein Asr and AFI help LF82 survival and replication in macrophages. Second, acid promotes the upregulation of nitrate metabolism genes, helping LF82 utilize nitrate in host cells for survival and replication. Third, acid downregulates the expression of flagellar genes, and LF82 shuts down flagellar biosynthesis after entering macrophages to save unnecessary energy loss to support intracellular survival and replication.

## 4 Materials and methods

### 4.1 Bacterial strains, plasmids, and growth conditions

The bacterial strains and plasmids used in this study are listed in **Table S2**. *E. coli* LF82 O83:H7 was used as the WT strain. Mutant strains were generated using the λ Red recombinase system of pSim6 and primers carrying the 39~45 bp homologous regions flanking the start and stop codons of the gene to be deleted, as previously described (Liu et al., 2019). Plasmid pUC57 carrying red fluorescent protein (mCherry) was used for confocal microscope observation and analysis. Plasmid vectors pSWK129 were used for complementation.Bacteria were cultured overnight at 37°C in LB liquid or LB agar plate. When necessary, appropriate antibiotics were added: ampicillin, 50 μg/ml; kanamycin, 50 μg/ml; chloramphenicol, 25 μg/ml; gentamicin, 20 μg/mL or 100 μg/mL.

### 4.2 Transcriptome response of LF82 to Acid

The LF82 glycerin tube frozen in the refrigerator at -80°C was frozen on ice, inoculated in LB liquid medium at a ratio of 1:1000 for overnight culture (about 16 hours), then inoculated in LB liquid medium at a ratio of 1: 100 for logarithmic phase and centrifuged at 5,500 rpm for 5 min. The supernatant was discarded to collect the bacteria and washed with 1640 (containing 10% FBS (fetal bovine serum)) medium 3 times. The precipitate of the bacteria was suspended in 1640 (containing 10% FBS, pH=5.8, the pH was adjusted to 5.8 with HCl) medium as acid treated group and 1640 (containing 10% FBS, pH=7.5) as control group, cultured in a shaking table at 37°C for 180 rpm for 30 min, and centrifuged at 5500 r for 5 min to collect the precipitate.

#### 4.2.1 RNA extraction and transcriptome sequencing

The cell wall of bacteria was broken by ultra-low-temperature grinding with liquid nitrogen, repeated 3~5 times until samples turned to powder; TRIzol was added (according to 10^7^ cell/mL TRIzol) and then gound to powder. Total RNA was extracted according to the manufacturer’s instructions of TRIzol-chloroform extraction (Invitrogen, California, USA). The concentration and purity of the extracted RNA were detected by Nanodrop 2000, the integrity of RNA was detected by agarose gel electrophoresis, and RIN (RNA integrity number) were detected by Agilent2100. To ensure that the single database construction met the requirements of total RNA amount of 2 μg, concentration was ≥ 100 ng/μL and OD_260/280_ was between 1.8 and 2.2.

rRNA was removed from total RNA; the mRNA was randomly broken into about 200 bp small fragments by adding the fragmentation buffer and used for first-strand cDNA synthesis by using reverse transcriptase and random primers. dUTP was used instead of dTTP in the dNTP reagent to ensure the base in the second strand of cDNA containing A/U/C/G. The cDNA library was constructed using extracted mRNA by the Truseq™ RNA sample prep kit (Illumina, California, USA). Before PCR amplification, the second strand of cDNA was digested by UNG enzyme, so that the library only contained the first strand of cDNA. Sequencing was carried out on Illumina HiSeq 2000 platform located in Majorbio Bio-Pharm Technology Co., Ltd. (Shanghai, China), and the original reading was generated.

#### 4.2.2 Analysis of differentially expressed genes

The original data after quality control, namely, clean data (reads), were compared with the reference genome to obtain mapped data (reads) for subsequent analysis. Meanwhile, the quality of the comparison results of transcriptome sequencing was evaluated, mainly including sequencing saturation, gene coverage, distribution of reads in different regions of the reference genome and distribution analysis of reads in different chromosomes. After obtaining qualified gene readings, the differential expression analysis of genes among samples was carried out. The gene expression difference between the acid-treated sample and the control sample was compared, and the mRNA abundance was estimated by the unique mapping reading number per thousand bases per million reading (RPKM) method (Bullard et al., 2010; Liu et al., 2017). The error rate (FDR) controls the true number used to obtain DEG (Audic and Claverie, 1997). The functional classification of DEGs in transcriptome was analyzed by evolutionary genealogy of genes: Non-supervised Orthologous Groups (EggNOG), Gene Ontology (GO), and Kyoto Encyclopedia of Genes and Genomes (KEGG) databases.

### 4.3 Growth curve

To determine the growth curve of each strain, overnight cultures were washed with PBS 3 times and diluted (1:1000) in LB without antibiotics. A 200 μL aliquot was added to a 96-well flat-bottom microplate, and 200 μL LB medium was used as negative control and incubated at 37 °C with shaking at 180 rpm for 24 h, as previously described (Liu et al., 2021). The absorbance at 600 nm was recorded. Experiments were independently performed three times.

### 4.4 Cell culture and macrophage replication

Raw 264.7, a murine macrophage cell line, 5×10^5^/well in 12-well cell culture plate was cultured with RPMI 1640 (containing 10% FBS and 1% penicillin and streptomycin (P/S) when necessary) medium at 37°C in 5% CO_2_. Bacterial uptake, survival, and replication were measured by gentamicin protection test. Before infection, the bacteria were washed with PBS and resuspended in RPMI 1640 and incubated at 37°C with shaking at 180 rpm for 30 min, infected 5×10^7^/well with multiplicity of infection (MOI) of 100∶1, centrifuged at 1,000× g for 10 min, incubated with 5% CO_2_ at 37°C for 10 min, and then extracellular bacteria were killed with 100 μg/ml gentamicin for 40 min (defined as T1). Ammonium chloride (NH_4_Cl, Sigma) and chloroquine diphosphate (CQ, APExBIO) were used for acid neutralization to study the effect of pH on the replication of LF82 in macrophages.; Na_2_NO_3_ was added to RPMI 1640 to study the effect of nitrate on the replication of LF82 in macrophages. In order to determine the number of bacteria in cells, 1% triton X-100 was added to each well for 5 min to lyse eukaryotic cells. Triton X-100 at this concentration has no effect on bacterial viability for at least 30 min. Samples were diluted and spread on LB agar plates to determine the amount of CFU recovered from the cracked monolayer. T24/T1 is the replication multiple of gentamicin treatment for 24 hours compared with gentamicin treatment for 1 hour.

### 4.5 Quantitative RT-PCR

The complementary DNA (cDNA) was generated from 1 μg of total RNA using the Primescript 1st strand cDNA synthesis kit (Takara, Shiga, Japan). The cDNA sample was diluted three-fold prior to performing downstream experiments. qRT-PCR was performed using the Applied Biosystems 7500 real-time PCR system and SYBR green PCR master mix (Applied Biosystems, Waltham, MA, USA). All the data were normalized to levels of housekeeping gene 16S (Tasara and Stephan, 2007). The relative expression level of each gene was calculated using the cycle threshold method (2^−ΔΔCt^) (Livak and Schmittgen, 2001). At least three biological replicates were carried out for each experiment. All the oligonucleotides used for qRT-PCR are listed in **Table S3**.

### 4.6 Adherence to epithelial cell experiment

Overnight bacteria were subcultured in DMEM (containing 10% FBS) medium at 37°C until OD_600_ ≈ 0.6-0.8. HeLa cells were washed with PBS three times before infection. The cell culture medium was replaced with fresh DMEM without antibiotics or FBS. Then, the cells were infected with bacteria cultured in DMEM with MOI of 100∶1. After incubation with HeLa cells for 3 hours (Duan et al., 2013), unattached bacteria were removed by washing with PBS 6 times. Then, HeLa cells were lysed with 0.1% SDS (H_2_O as solvent). The lysate was spread on an LB agar plate to count the number of surviving adherent bacteria. In order to determine the number of intracellular bacteria, fresh cell culture medium containing 100 g/mL gentamicin (Sigma) was added for 1 hour to kill extracellular bacteria. Cells were then lysed with 1% Triton X-100, and bacteria were quantified as described above.

### 4.7 Motility assays

Bacteria were cultured overnight in LB liquid medium at 37°C and 180 rpm, and 2 μL of culture was inoculated on 0.3% LB agar plate. The plate was cultured at 37°C for 9 h, and the motility was quantitatively evaluated by examining the circular swimming movement of the growing active bacterial cells.

### 4.8 Confocal observation

After infection with bacteria containing mCherry plasmid, cells were washed with PBS to eliminate non-adsorbed bacteria or extracellular bacteria, and fixed with 3% paraformaldehyde for 10 min. Subsequently, the cells were washed with PBS, washed with PBS, and permeabilized with 0.1% Triton X-100 for 20 min. After washing with PBS, the slides were incubated with PBS-0.2% gelatin twice for 10 min each time. Then, the monolayer was washed with PBS and distilled water, and the stationary solution was fixed on the glass slide. The coverslips were mounted on slides and cell images were acquired using a confocal laser scanning microscope (Zeiss LSM800) and analyzed with ZEN 2.3 (blue edition) and format design by Adobe Illustrator CC 2018 softwares. Data is based on the clearest results preserved from five fields of three slides.

### 4.9 Statistical analysis

Statistical analysis was conducted using GraphPad Prism software (v9.3.0; GraphPad Software, San Diego, CA, USA). The mean ± SD from three independent experiments is shown in the figures. Differences between two mean values were evaluated using a two-tailed Student’s *t*-test. Statistical significance was set at *P* < 0.05.

## Data availability statement

The datasets presented in this study can be found in online repositories. The names of the repository/repositories and accession number(s) can be found below: NCBI, BioProject PRJNA982418.

## Author contributions

TY: Methodology, Software, Writing – original draft, Writing – review & editing. YH: Validation, Writing – review & editing. ZH: Validation, Writing – review & editing. XML: Validation, Writing – review & editing. XWL: Validation, Writing – review & editing. YL: Conceptualization, Data curation, Funding acquisition, Project administration, Resources, Supervision, Writing – original draft, Writing – review & editing. HS: Software, Writing – review & editing. YP: Funding acquisition, Project administration, Writing – review & editing.
