# Supplementary material for: Response mechanisms to acid stress promote LF82 replication in macrophages
Source: Front Cell Infect Microbiol. 2023 Oct 10;13:1255083. doi: 10.3389/fcimb.2023.1255083 (PMC10595154; doi:10.3389/fcimb.2023.1255083)
Supplement: Supplementary file 2 [file DataSheet_2.pdf]

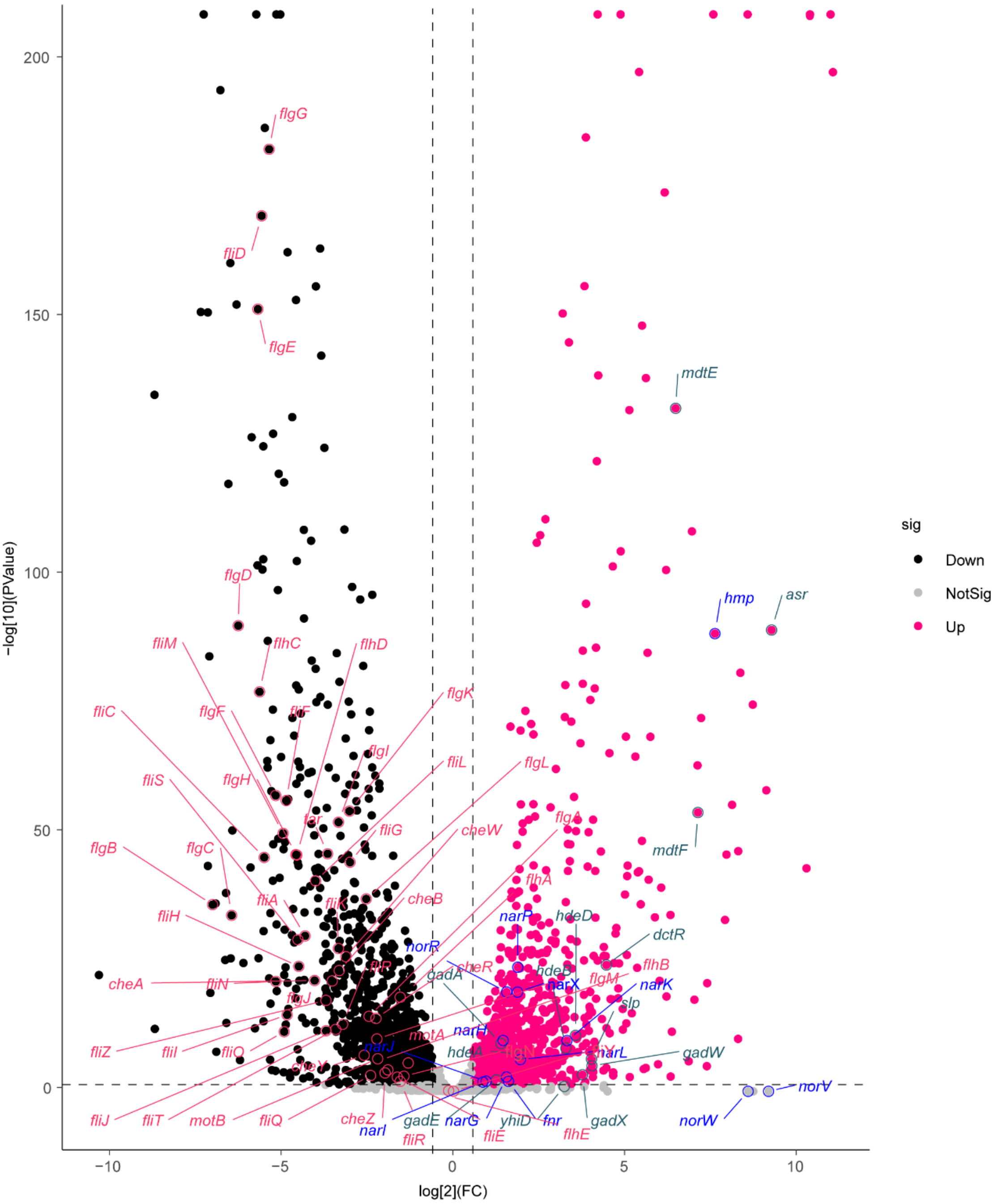

Figure S2 (A) Genes of acid, nitrate metabolism and flagellar were marked in volcano plot.

Table S3 Viable bacteria of WT and mutants of LF82 treated with or without NH_4Cl and CQ at 1 h p.i. and 24 h p.i. within Raw 264.7 macrophages.

Strains	10^5CFU/Well		
	T1	T24	T24/T1
WT	7.47±0.95	25.2±3.34	3.38±0.2
Δ*asr*	7.27±1.33	6.5±1.65	0.89±0.07
Δ*asr*+p*asr*	7.83±1.65	25.23±4.63	3.26±0.41

Δ*gadA*	8.2±0.72	7.89±1.22	0.96±0.06
Δ*gadA*+p*gadA*	8.3±1.05	25.83±2.1	3.15±0.5
Δ*hdeA*	10.93±0.5	16.57±1.8	1.52±0.17
Δ*hdeA*+p*hdeA*	9.03±1.46	28.97±7.5	3.17±0.34
Δ*hdeB*	9.6±1.06	13.86±2.64	1.44±0.14
Δ*hdeB*+p*hdeB*	10±1.04	31.2±1.65	3.13±0.16
Acid neutralization	T1	T24	T24/T1
WT-NH_4Cl	6.2±0.2	6.29±0.43	1.01±0.04
Δ*asr*-NH_4Cl	6.73±0.23	6.74±0.47	1±0.04
Δ*gadA*-NH_4Cl	8.6±0.92	7.15±1.15	0.84±0.15
Δ*hdeA*-NH_4Cl	9.8±0.4	10.3±0.35	1.05±0.04
Δ*hdeB*-NH_4Cl	9.27±0.42	9.72±0.91	1.05±0.06
WT-CQ	4.53±0.95	3.67±0.69	0.81±0.02
Δ*asr*-CQ	3.8±0.69	2.85±0.3	0.76±0.09
Δ*gadA*-CQ	7±0.69	5.96±0.66	0.86±0.15
Δ*hdeA*-CQ	8±1.4	6.41±1.06	0.8±0.01
Δ*hdeB*-CQ	8.4±1.4	7.02±1.27	0.84±0.02

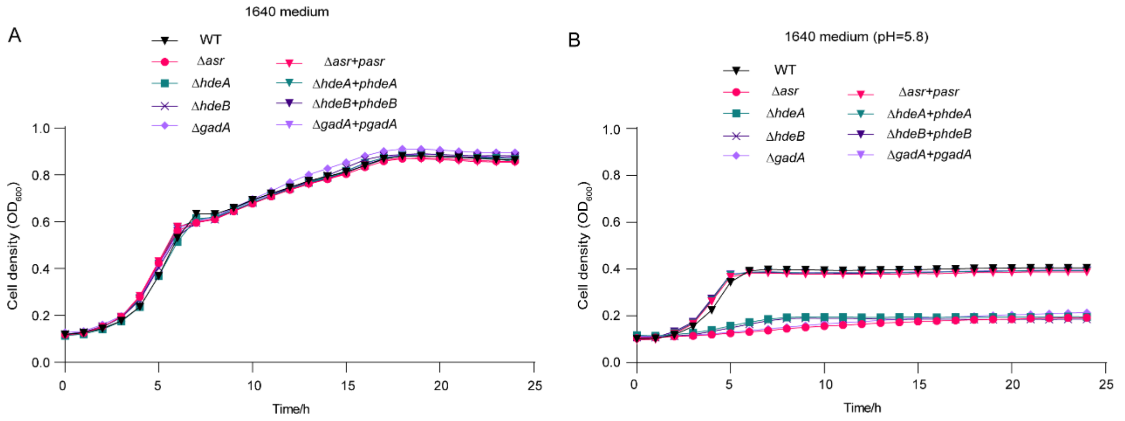

Figure S3 (A) Growth curve of Δ*asr*, Δ*asr*+p*asr*, Δ*gadA*, Δ*gadA*+p*gadA*, Δ*hdeA*, Δ*hdeA*+p*hdeA*, Δ*hdeB*, Δ*hdeB*+p*hdeB* and WT in 1640 medium. (B) Growth curve of Δ*asr*, Δ*asr*+p*asr*, Δ*gadA*, Δ*gadA*+p*gadA*, Δ*hdeA*, Δ*hdeA*+p*hdeA*, Δ*hdeB*, Δ*hdeB*+p*hdeB* and WT in 1640 medium (pH=5.8).

Table S4 Viable bacteria of WT and mutants of LF82 treated with or without nitrate at 1 h p.i. and 24 h p.i. within Raw 264.7 macrophages.

Strains	10^5CFU/Well		
	T1	T24	T24/T1
WT	6.87±0.58	21.37±1.05	3.12±0.15
Δ*narXL*	7.93±0.83	8.23±1.07	1.04±0.04
Δ*narXL*+p*narXL*	7.3±1.11	22.93±2.88	3.15±0.12
Δ*nark*	6.8±0.87	8.57±0.72	1.26±0.05
Δ*narK*+p*narK*	6.37±0.91	20.13±2.92	3.16±0.07
Δ*hmp*	6.47±1.17	5.57±1.42	0.85±0.06
Δ*hmp*+p*hmp*	6.47±1.22	19.77±5.15	3.03±0.22
Δ*narP*	6.63±1.83	7.55±2.44	1.13±0.06
Δ*narP*+p*narP*	7.13±1.8	21.5±4.33	3.04±0.15
Nitrate	T1	T24	T24/T1
WT	5.33±0.81	54±7.55	10.14±0.12
Δ*narXL*	6.73±0.99	6.97±1.25	1.03±0.07
Δ*narXL*+p*narXL*	6.2±1.25	63.67±4.25	10.47±1.64
Δ*nark*	6.33±1.21	8.17±0.86	1.3±0.11
Δ*narK*+p*narK*	6.73±1.34	69.33±2.08	10.57±2.03
Δ*hmp*	7.6±2.62	6.7±2.19	0.89±0.03
Δ*hmp*+p*hmp*	6.63±0.57	69.67±1.53	10.54±0.7
Δ*narp*	5.67±0.61	6.73±0.42	1.19±0.08
Δ*narP*+p*narP*	7.2±2.55	71.17±5.84	10.47±2.49

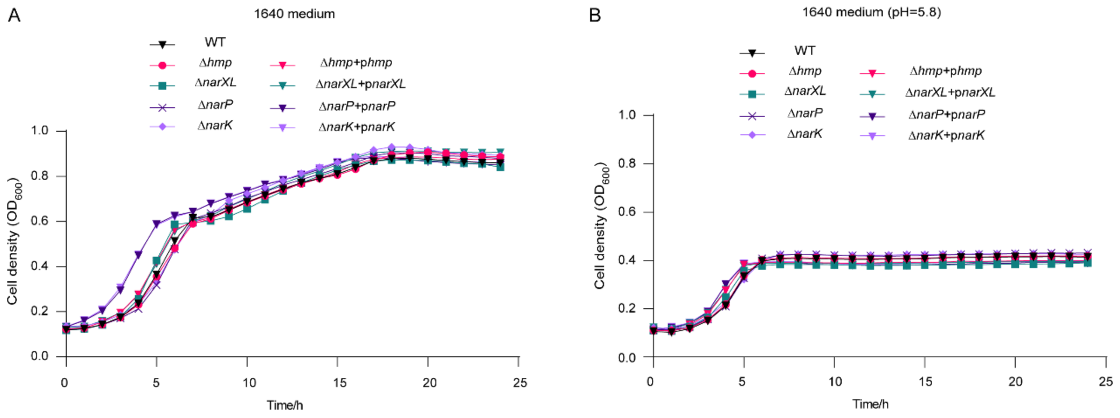

Figure S4 (A) Growth curve of *narK*, Δ*narK*+p*narK*, *narXL*, Δ*narXL*+p*narXL*, *narP*, Δ*narP*+p*narP*, *hmp*, Δ*hmp*+p*hmp* and WT in 1640 medium. (B) Growth curve of *narK*, Δ*narK*+p*narK*, *narXL*, Δ*narXL*+p*narXL*, *narP*, Δ*narP*+p*narP*, *hmp*, Δ*hmp*+p*hmp* and WT in 1640 medium (pH=5.8).

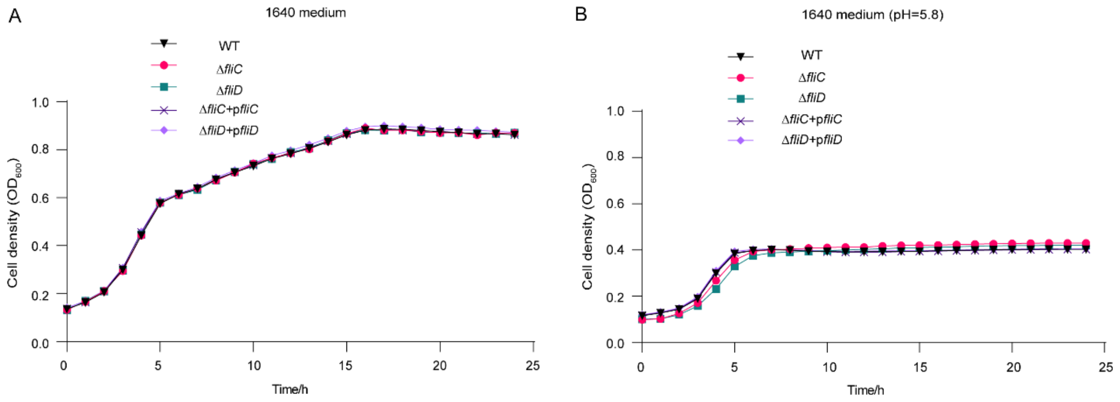

Figure S5 (A) Growth curve of Δ*fliC*, Δ*fliD* and WT in 1640 medium. (B) Growth curve of *fliC*, *fliD* and WT in 1640 medium (pH=5.8).

Table S5 Viable bacteria of WT and Δ*fliC*, Δ*fliD* mutants of LF82 at 1 h p.i. and 24 h p.i. within Raw 264.7 macrophages.

Strains	10^5CFU/Well		
	T1	T24	T24/T1
WT	8.47±1.32	26.67±3.55	3.16±0.1
Δ*fliC*	8.3±1.06	22.81±0.23	2.78±0.35
Δ*fliD*	7.93±1.31	23.95±1.36	3.05±0.32
