# Supplementary material for: Response mechanisms to acid stress promote LF82 replication in macrophages
Source: Front Cell Infect Microbiol. 2023 Oct 10;13:1255083. doi: 10.3389/fcimb.2023.1255083 (PMC10595154; doi:10.3389/fcimb.2023.1255083)
Supplement: Supplementary file 1 [file DataSheet_1.zip › Supplementary/Table S2.doc]

**Table S2. Strains and plasmids used in this study**

| **Strains** | **Genotype or description** | **Source** |
| --- | --- | --- |
| DH5α | A kind of *Escherichia coli* widely used for gene cloning | Our lab |
| BL21 | A kind of *Escherichia coli* widely used for expression of recombinant proteins | Our lab |
| WT LF82 | Wild-type adherent–invasive *E. coli* LF82 O83:H7 | Our lab |
| Δ*asr* | *asr* deletion mutant in LF82 | This study |
| Δ*gadA* | *gadA* deletion mutant in LF82 | This study |
| Δ*hdeA* | *hdeA* deletion mutant in LF82 | This study |
| Δ*hdeB* | *hdeB* deletion mutant in LF82 | This study |
| Δ*narK* | *narK* deletion mutant in LF82 | This study |
| Δ*narXL* | *narXL* deletion mutant in LF82 | This study |
| Δ*narP* | *narP* deletion mutant in LF82 | This study |
| Δ*hmP* | *hmP* deletion mutant in LF82 | This study |
| Δ*fliC* | *fliC*deletion mutant in LF82 | This study |
| Δ*fliD* | *fliD* deletion mutant in LF82 | This study |
| **Plasmids** | | |
| pSim6 | λ Red recombinase system | Our lab |
| pKD3 | Containing a chloramphenicol resistance cassette BfR†  and the flipase recognition sites，CmR | Our lab |
| pKD4 | Containing a kanamycin resistance cassette and the flipase recognition sites，KmR | Our lab |
| pUC57 | Containing a red fluorescent protein (mCherry) | This study |
