# Supplementary material for: Response mechanisms to acid stress promote LF82 replication in macrophages
Source: Front Cell Infect Microbiol. 2023 Oct 10;13:1255083. doi: 10.3389/fcimb.2023.1255083 (PMC10595154; doi:10.3389/fcimb.2023.1255083)
Supplement: Supplementary file 1 [file DataSheet_1.zip › Supplementary/Table S3.doc]

**Table S2. Strains and plasmids used in this study**

| **Strains** | **F/R** | **Sequence** |
| --- | --- | --- |
| **Primers for WT identifying** | | |
| wzx | F | CGCTCTAATGAACTACTGTCTCC |
| wzx | R | TTTTCCGTGATTTCCCTTG |
| wzy | F | GTTTTAATCTGGTTTTCGCTACC |
| wzy | R | TGCTGTTTTACAAGTTTCTGCA |
| **Primers for gene mutation** | | |
| *asr* | F | cagtggggttaaatgaaaaaacaaattgagggtatgacaGTGTAGGCTGGAGCTGCTTC |
| *asr* | R | tcaggcgcgagggggcgcgccagcattactgttgaaaacATGGGAATTAGCCATGGTCC |
| *gadA* | F | tttaaaggttgggcattcggtttttacaacgtgatgttaATGGGAATTAGCCATGGTCC |
| *gadA* | R | acttttttattgccttcaaataaatttaaggagttcgaaGTGTAGGCTGGAGCTGCTTC |
| *hdeA* | F | tcaatatgcaaggaaatacgatgtaacgcattcccggaaATGGGAATTAGCCATGGTCC |
| *hdeA* | R | tcgtgacggctctttcactttatagttgaggatattacgGTGTAGGCTGGAGCTGCTTC |
| *hdeB* | F | cctgtttgtatataatctctatattgaatgggttacaaaGTGTAGGCTGGAGCTGCTTC |
| *hdeB* | R | agatggctcaacttgctactcctttttacttgtaccttaATGGGAATTAGCCATGGTCC |
| *narK* | F | cagacactaaggtggcagacatcgaaacgagtatcagaggtgtctGTGTAGGCTGGAGCTGCTTC |
| *narK* | R | aaagcgcgacatcatgccgcgcaaaggataatcaaatattacgatATGGGAATTAGCCATGGTCC |
| *narXL* | F | ccgttcgacgcattgtcaaacgacgaactgcgctgggaaccgtaaGTGTAGGCTGGAGCTGCTTC |
| *narXL* | R | tcattaaggttattgctcatttaaagcctgaaggaagaggtttacATGGGAATTAGCCATGGTCC |
| *narP* | F | acattcacgcgcctggtagcgttaccaacgctacgctcaaacataGTGTAGGCTGGAGCTGCTTC |
| *narP* | R | agagtaaataaaaagtggcccgatggtgatgccatcgggctatttATGGGAATTAGCCATGGTCC |
| *hmP* | F | tgcatttgagatacatcaattaagatgcaaaaaaaggaagaccatGTGTAGGCTGGAGCTGCTTC |
| *hmP* | R | aaacgggtcaagggttgccggatgtgtccatccggcaacatcaaaATGGGAATTAGCCATGGTCC |
| *fliC* | F | gcaatttggcgttgccgtcagtctcagttaatcaggttacggcgaGTGTAGGCTGGAGCTGCTTC |
| *fliC* | R | aaacccaaaacgtaatcaacgacttgcaatataggataacgaatcATGGGAATTAGCCATGGTCC |
| *fliD* | F | aaccccggtattcgttttacgtgtcgaaagataaaaggaaatcgcGTGTAGGCTGGAGCTGCTTC |
| *fliD* | R | ggtgccttttgccgcgtacatgacctgtctcccgatgaatattgaATGGGAATTAGCCATGGTCC |
| **Primers for identifying the gene mutants** | | |
| *asr* | F | CCGGACTTATTGCCTGCTCA |
| *asr* | R | TGCACCCAACACTACAGCAA |
| *gadA* | F | CGGGCTGGATGACTGGA |
| *gadA* | R | GGAGTGTTTGGGCGATTT |
| *hdeA* | F | CGCAACTGGTGTCATTGCTT |
| *hdeA* | R | GATACACAGCAACCCGACGA |
| *hdeB* | F | CGACGATAAACAGCAGCACG |
| *hdeB* | R | CTTTCCATGCCAATAGCGCC |
| *narK* | F | ATTTGCCGTGTGGTTAGTCG |
| *narK* | R | TAAGGGCAAGGTAACGCTCTG |
| *narXL* | F | AACTGATTTGGCAGGGCGAT |
| *narXL* | R | CATCCATACGCAAAACGCCA |
| *narP* | F | AACGCTGTAGCCACCAGAAC |
| *narP* | R | CTGCACTGGGATACGCTGAA |
| *hmP* | F | TGGACAACGGCGAACAGTAT |
| *hmP* | R | CGGTGCGGAGTATATGGTGG |
| *fliC* | F | CGCCTGTAGCAGATCGTCAA |
| *fliC* | R | CCGACTCCCAGCGATGAAAT |
| *fliD* | F | CAAGCCAGAAGACAGACGCT |
| *fliD* | R | AACAGCGTGGGATTACGCAT |
| **Primers for qRT-PCR** | | |
| *asr* | F | ATTAGCTCTGGTTGTTGCCG |
| *asr* | R | GGGGCTTTCTGTTCAGCTTTG |
| *gadA* | F | CTTCCTGGCACCGTTCGT |
| *gadA* | R | CGGGCGGGAGAAGTTGAT |
| *hdeA* | F | TTAGGCGTTATTCTTGGTGGTC |
| *hdeA* | R | CACAGCCAGGAAATCTTCACAG |
| *hdeB* | F | AGCCAATGAATCCGCTAAAGA |
| *hdeB* | R | AAAGTAACGGTATCGCCACCT |
| *narK* | F | AGCAGTTGCCGGTACTCAAA |
| *narK* | R | GCCCGAAGAAAGCGTATTGC |
| *narX* | F | GCTTACAGATGCAGGGCGAT |
| *narX* | R | CGTAATCCAGGCTCGGTGAG |
| *narL* | F | GCTGCTGGCGAAATGGTATTA |
| *narL* | R | TCATCTTGTTCGGCAAACCCT |
| *narP* | F | ACGCGGTGTTCGTCAGTTAC |
| *narP* | R | TTGAGGGTATCCAGGCCACT |
| *hmP* | F | CCTATGGCGTCCTGGCTAAT |
| *hmP* | R | AGCTGGTGATAAGTGCGCTG |
| *fliC* | F | ACCATTAGCGACCTGACAGC |
| *fliC* | R | TAGACCGTGGCAGTTTGAGC |
| *fliD* | F | GATGACACTCTCGGTCAGCG |
| *fliD* | R | CTGCTGTTCTCAATGGCGAC |
| *ytfE* | F | GTGCCAAAAGGGCTGACAAA |
| *ytfE* | R | CTTCATCGTGCTCGCTTTCC |
| *yeaR* | F | ATAAACAAACTGCGCCTGCC |
| *yeaR* | R | AACTGCCCCGCTTCGATAAG |
| *ibpB* | F | CCCGCCGTACAACATTGAGA |
| *ibpB* | R | TAAGCCCTTGATGCAGCCAT |
| *marR* | F | ACGAGTATCTGTCTCCGCTG |
| *marR* | R | ATCCAGCATACGGGTCAGTG |
| *tnaA* | F | AGATCACCCGCGAAACCTAC |
| *tnaA* | R | GCAAAGGGTTCTGCATTCGG |
| *srlE* | F | GGCGCAGTACATTGTGGAAG |
| *srlE* | R | CGGCAGAACCCATACCCATT |
| *mglA* | F | GTTACCGTATTGCGCGACG |
| *mglA* | R | CGTCAGGTTACGCACTTCCA |
| *argT* | F | ACCGATAAACGTCAGCAGGA |
| *argT* | R | GTTAGCGTATGCTTCCTGGGT |
| *gutM* | F | TATCCGCACTCATCACCGTC |
| *gutM* | R | GCTGATCGTCCAGCGCAATA |
| *nupG* | F | TTTGCTCTGTTTGCTTACGGC |
| *nupG* | R | CATCAGGAACATCCCCTGCG |
